# Substrate and Excitation Intensity Dependence of Saturable Absorption in Perovskite Quantum Dot Films

**DOI:** 10.3390/nano13050871

**Published:** 2023-02-26

**Authors:** Mithun Bhowmick, Bruno Ullrich, Madeline Murchland, Xuan Zhou, Chari Ramkumar

**Affiliations:** 1 Department of Mathematical and Physical Sciences, Miami University, 4200 N University Blvd., Middletown, OH 45042, USA; 2 Ullrich Photonics LLC, Manistique, MI 49854, USA; 3 Department of Geology and Environmental Science, Miami University, 250 S Patterson Ave., Oxford, OH 45056, USA; 4 Department of Physics and Astronomy, University of Texas at San Antonio, One UTSA Circle, San Antonio, TX 78249, USA; 5 Department of Physics, Geology & Engineering Technology, Northern Kentucky University, Nunn Drive, Highland Heights, KY 41099, USA

**Keywords:** perovskite, quantum dot, saturable absorber, photoluminescence

## Abstract

Saturable absorption in perovskite quantum dot (PQD) films, leading to saturation in photoluminescence (PL), is reported. PL of drop-casting films was used to probe how excitation intensity and host–substrate influence the growth of PL intensity. The PQD films were deposited on single-crystal GaAs, InP, Si wafers and glass. Saturable absorption was confirmed through PL saturation in all films, with different excitation intensity thresholds, suggesting strong substrate-dependent optical properties, resulting from absorption nonlinearities in the system. The observations extend our former studies (Appl. Phys. Lett., 2021, 119, 19, 192103), wherein we pointed out that the PL saturation in QDs can be used to create all-optical switches in combination with a bulk semiconductor host.

## 1. Introduction

Hybrid materials such as organolead halide perovskites have attracted significant attention for their high carrier mobilities and mechanical strength [1]. The general stoichiometry of such materials is APbX_3_ (A = organic ammonium cation, X = halide ion). These perovskites are attractive for their excellent optoelectronic properties [2,3,4]. Considering the economic viability of their synthesis, the bromide and iodide versions of the perovskites have shown great potential for photovoltaics [4]. 

The quest for novel materials for optoelectronic applications has led to fascinating structures based on quantum confinement. Colloidal quantum dots (QDs) are one of the most interesting materials, where narrow linewidths and high quantum yields are present with great wavelength tunability [5], whereas, out of the group, perovskite quantum dots (PQDs) themselves are one of the most important nano-sized arrangements due to their ability to bring great advantages for solar cell applications by producing inexpensive yet highly efficient photosensitive films [4,5,6].

Due to excellent luminescence properties, PQDs are also attractive for the formation of light-emitting devices (LEDs) and lasers [6]. The progress in developing PQD-based LEDs has been rapid, with recent reports of more than 21% for the external quantum efficiency (EQE) [5,6]. However, to become a viable alternative to traditional light-emitting devices (LEDs), EQE in PQD-based LEDs needs to be improved [4,5,6]. Further, the long-term stability needs significant improvement, especially in ambient atmospheres [5]. Finally, the ideal material will have to be environmentally acceptable to seamlessly utilize it in ambient-friendly technologies. These developments call for a detailed understanding of how PQDs respond to photoexcitation, specifically at high excitation intensities, where photoinduced damage could occur, causing dramatically reduced efficiency.

It is, thus, critical to investigate interactions of PQDs with coherent and incoherent light to explore possible optical regimes where novel applications could be harnessed. Recent reports have highlighted saturable absorption from quantum dots deposited on a GaAs substrate [7,8]. The same report also demonstrated a mutual alteration in photoluminescence (PL) properties of the QDs by GaAs [7]. Saturable absorption, stemming from nonlinear effects in QD systems, has been reported before, and is extremely attractive for potential applications in quantum information processing, cavity solitons and electromagnetically induced transparency, to mention a few [9,10,11,12]. In the context of the current work, saturable absorption refers to reduced absorption in emitters at higher excitation intensities. There are open questions regarding the mechanism of saturation of absorption reported before, for example, whether the substrate influences saturation trends, and whether all QDs demonstrate a similar mathematical trend in PL intensity growth with the excitation intensity increase [11,12,13].

To answer these open questions, a systematic PL study of PQDs was carried out herein, i.e., PL spectra were measured from PQD films deposited on four different substrates: glass, GaAs, InP and Si wafer. Hereafter, the four samples will be referred to as PV/glass, PV/GaAs, PV/InP and PV/Si, respectively.

## 2. Materials and Methods

**Figure 1:**
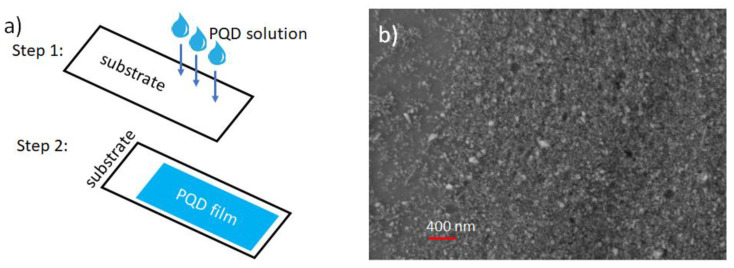
(**a**) Schematic diagram showing the drop-casting recipe to deposit PQD films on the substrates; (**b**) SEM image of the deposited PQD film on glass.

One-sided polished, <100> oriented single-crystal GaAs, InP and Si substrates from University Wafers Inc. (South Boston, MA, United States) were used for sample preparation. GaAs and InP wafers were undoped (thickness: 300 µm), while the Si wafer possessed p-type doping (thickness: 525 µm ± 25 µm). Oleic acid and oleylamine-coated PQD (FAPbBr_3_) solution in toluene from Sigma Aldrich (St. Louis, MO, United States) were used without further processing. The QD solutions came with manufacturer’s specifications of a quantum yield ≥ 70%, emitting between 525 and 535 nm wavelength, with full width at half maximum (FWHM) ≤ 30 nm. The solution was then drop casted on the substrates: each time, 1 mL of the solution was uniformly poured on the respective host (step 1 of the process, as shown in Figure 1a), waiting for 120 min, which was roughly the time required for the PQD solution to form a solid film on the substrates (step 2 of the process, as shown in Figure 1a). The glass substrate was pre-cleaned using acetone and ethanol prior to deposition. The wafers were cleaned using ethanol before the deposition of films on their polished surfaces. A scanning electron microscope (SEM) from Zeiss (White Plains, NY, United States) was employed to characterize the PV/glass sample. The image confirmed deposition of the films and uniform presence of PQD, as presented in Figure 1b.

Optical profilometry was performed using a Local Electrode Atom Probe (LEAP) 5000XS from Cameca Instruments Inc. (Fitchburg, MA, United States) to determine thicknesses of the films, which were found to be 0.5 µm ± 0.17 µm for all samples. The effects of sample thickness were tested by making two more films on glass with 0.25 µm and 0.75 µm thicknesses. The results did not show any difference in PL linewidth and intensity growth.

**Figure 2:**
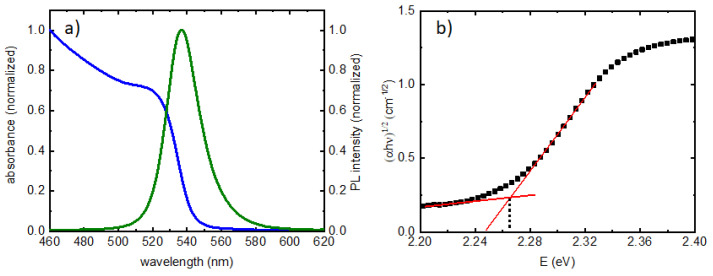
(**a**) Normalized absorbance and PL from the PQD film on glass; (**b**) determination of band gap from absorbance, corrected for tail states according to Ref. [14].

Absorption spectra and PL were collected from the PQD film on glass substrate to clarify the excitation/emission characteristics of the film, presented in Figure 2a. The absorption peak showed the 1st exciton at ~525 nm, while the emission wavelength was found to be 537 nm, with a FWHM confirmed to be ~23 nm.

The PL spectra from the deposited films were excited using a 405 nm, continuous-wave (CW) laser from Laserglow Technologies (North York, PA, United States) with a maximum power of 315 mW. The band gap was determined to be 2.27 eV using the absorbance spectra, as presented in Figure 2b and described previously [14]. All measurements were taken from the linear region of the output power vs. diode current curve. The output powers were monitored by a computer-controlled power meter from Laserglow Technologies (North York, Ontario, Canada) with 3% or less uncertainty due to shot-to-shot fluctuations. The laser output powers were then converted to intensity as follows:

intensity (W/cm^2^) = output power (W)/area of laser beam (cm^2^) (1)

where “output power” is defined as the power measured at the sample plane, and “Area” refers to the area covered by the laser beam at the same plane. Unless otherwise stated, exposure time was 5 s and area of the beam was estimated as 0.1257 cm^2^ for all measurements reported here.

PL spectra were collected in backscattering geometry using a home-built setup comprising a 450 nm-long pass filter and a focusing lens, for subsequent collection by a fiber-coupled spectrometer from StellerNet Inc. (Tampa, FL, USA). The spectra were collected without averaging and any integration time, and they were analyzed. All measurements were performed at room temperature (300 K).

## 3. Results

**Figure 3:**
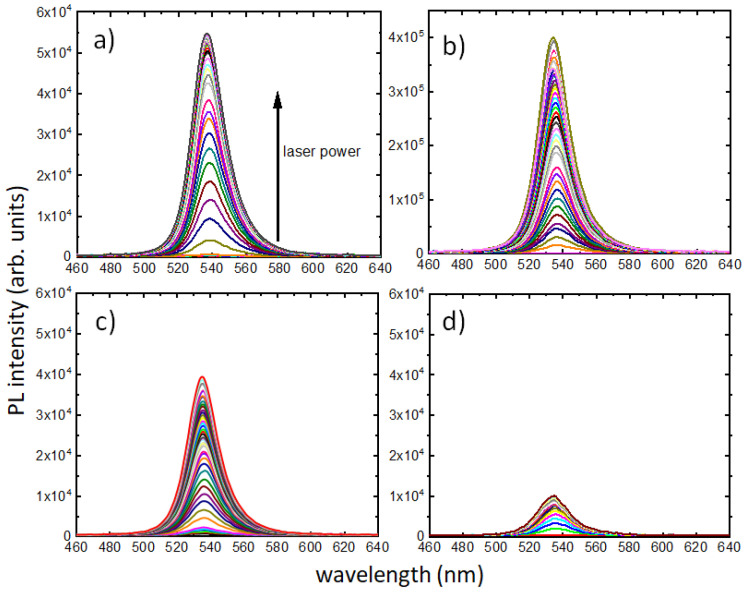
PL from (**a**) PV/glass, (**b**) PV/GaAs, (**c**) PV/InP and (**d**) PV/Si samples. The arrow indicates the direction of increment in laser power for (**a**–**d**). Note the significantly higher signal for PV/GaAs traces in (**b**).

**Figure 4:**
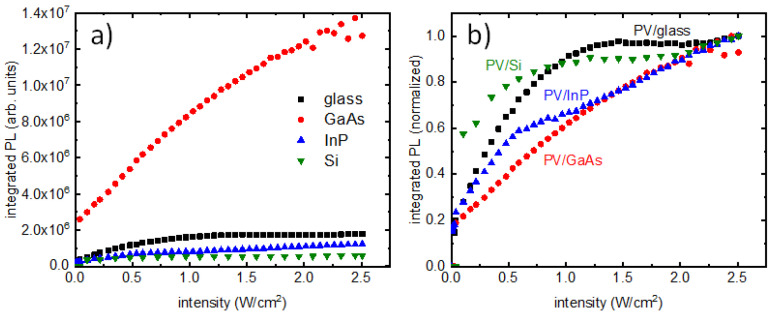
(**a**) Integrated PL from samples as a function of increasing intensity. (**b**) Normalized integrated PL from all samples as a function of increasing intensity.

Steady-state PL spectra were measured from the PQD films as a function of laser powers between 0 and 315 mW, with 5 mW intervals for all samples, except for the PV/Si sample, where the interval was 10 mW. All the PL measurements and corresponding growth of PL intensities with laser intensity are presented in Figure 3 and Figure 4, respectively. It is clear from Figure 3b that the PV/GaAs sample has PL intensity one order of magnitude higher than the rest of the samples and so normalization versions were needed for comparison. Figure 4a presents a comparison of PL intensity growth in all samples, with Figure 4b showing a comparison of their normalized versions. The different step sizes used in PV/Si measurements did not contribute to the trend, as is evident in Figure 4a. The minimum laser power for which an emission was detected for the PV/glass sample was 3.5 mW (Figure 3a), corresponding to an intensity of ~0.0278 W/cm^2^. The minimum excitation intensities for PL in PV/GaAs, PV/InP and PV/Si samples could be found from the respective plots as ~0.0278 W/cm^2^, ~0.0040 W/cm^2^ and ~0.1035 W/cm^2^. The thresholds of PL saturation for PV/glass, PV/GaAs, PV/InP and PV/Si samples could be found from Figure 4b to be at ~1.0 W/cm^2^, ~2.0 W/cm^2^, ~0.7 W/cm^2^ and ~0.5 W/cm^2^, respectively. These results confirm a universal saturation of emission in PQDs, where the thresholds of saturation are dependent on the substrates on which the films were deposited. In addition, there are differences in the way integrated PL evolved as laser intensity increased.

## 4. Discussion

In a previously published work, PbS quantum dots were studied to investigate interaction between nanoparticles and substrates [7]. The two-photon excitation measurements presented in that work, carried out using a pulsed excitation source, revealed dramatically saturating emission at high intensity values [7]. The PL measurements presented in this work clearly show the same features. This PL saturation can be employed to initiate all-optical switches in conjunction with luminescent substrates. The film absorbs impinging photons until the saturation thresholds, where it abruptly stops absorbing any light and becomes transparent. Notably, the saturation threshold varies for different substrates, as clearly presented in Figure 4. Thus, it is possible to tune the saturation behavior by simply changing the substrate, which could be extremely useful for device applications. While the reported results deserve a more theoretical analysis, the key in PL saturation seems to be the creation of one electron–hole pair per excited QD in the PQD film [13].

The steady-state PL for all samples has several notable features; a qualitative discussion is presented here to note the key observations. As seen in Figure 3, the PQD films did not emit light until a certain threshold of excitation. For PV/glass, PV/GaAs, PV/InP and PV/Si samples, this threshold was 3.5 mW (0.0278 W/cm^2^), 3.5 mW (0.0278 W/cm^2^), 0.5 mW (0.0040 W/cm^2^) and 13 mW (0.1035 W/cm^2^), respectively. Clearly, the PV/InP sample emitted significantly smaller excitation. It is also noteworthy that, compared to the other samples, PV/InP showed a markedly different trajectory for the growth of PL emission as the intensity increased. As seen in Figure 4b, for PV/InP, the initial flattening of the curve occurs at ~ 0.7 W/cm^2^ intensity. However, for higher excitation intensities, the PL intensity starts to change again at ~1.0 W/cm^2^ and increases almost linearly for the remainder of the intensity values. This is clearly different from the PV/glass sample, where the PL intensity saturates at ~ 1.0 W/cm^2^. For the PV/GaAs sample, this saturation happens at a much larger value of intensity (~2.0 W/cm^2^). Finally, in the case of the PV/Si sample, a clear saturation is seen at ~ 0.5 W/cm^2^.

**Figure 5:**
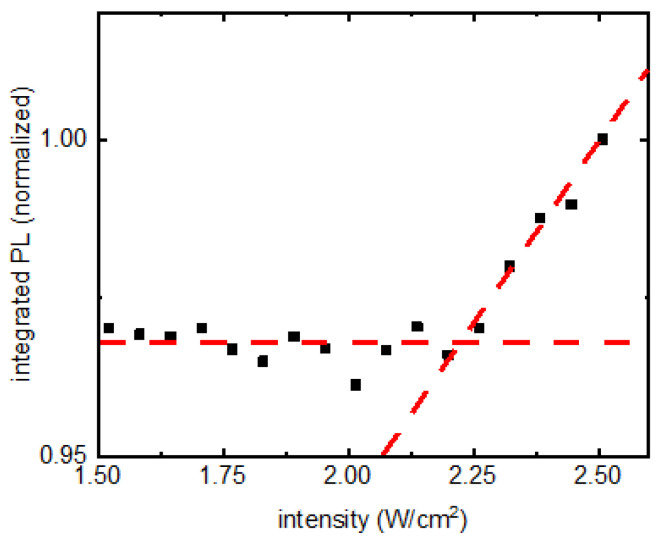
Transition of PL intensity in PV/glass into another linearly growing region shown.

A careful examination of Figure 4b for the final four points in the PV/glass and PV/Si trajectories reveals a change in slope in them. A zoomed-in version of that region from PV/glass is presented in Figure 5, showing the transition. This could indicate the presence of a region where PL would grow linearly for these two samples as well. The second phase of increasing PL intensity could either be material damage due to long exposure of high CW intensity or could be due to nonlinear effects in nanomaterials. Laser damage of materials was ruled out by repeating the experiments revealing the same results.

Considering the similarity among the deposited PQD films in thickness and uniformity, it is highly unlikely that the difference in PL saturation stems from the intrinsic properties of the films themselves. Rather, it appears that the change in threshold is due to the interaction of PQD films with the substrates. It was previously shown that GaAs significantly alters emission properties of quantum dot systems [7]. However, reproducing the same with reasonably low intensity is a noteworthy development. The saturation of PL intensity, due to saturation of absorption with CW excitation, was previously reported in quantum-dot-based saturable absorption mirrors [10,11,12,15]. The saturation of absorption was attributed to nonlinear refractive index of the quantum dot ensembles [11,15].

To summarize, steady-state PL measurements were conducted in PQD films deposited on four different substrates. All samples showed saturation of emission at different thresholds of intensities. The saturation of PQD samples at certain, characteristic intensity values is probably a result of creating all the electron–hole pairs possible in the QDs, at which point there are no more electrons to be excited. In addition, the absorption saturation might also be influenced by nonlinear optical properties [9,10,11,12] of the films at sufficient laser intensities. PQD-based saturable absorbers, employing a simple drop-casting deposition scheme, may lead to new devices where high quantum yield, narrow selectivity and low power consumption are critical. The variation in PL growth pathways and eventual saturation, as evidenced in this work, is remarkable and could easily be achieved through changing the substrate on which the films are deposited. Apart from saturation absorption, these results could potentially lead to the realization of optical pattern formation and spatial solitons (e.g., cavity solitons).

## Data Availability

The data presented in this study are available within the article.
